# Post-urethroplasty complications in hypospadias repair: a systematic review and meta-analysis comparing polydioxanone and polyglactin sutures

**DOI:** 10.1136/wjps-2023-000659

**Published:** 2024-03-01

**Authors:** Nitinkumar Borkar, Charu Tiwari, Debajyoti Mohanty, Tridip Dutta Baruah, Manoj Mohanty, C K Sinha

**Affiliations:** 1 Pediatric Surgery, All India Institute of Medical Sciences-Raipur, Raipur, Chhattisgardh, India; 2 General Surgery, All India Institute of Medical Sciences-Raipur, Raipur, Chhattisgardh, India; 3 Pediatric Surgery, All India Institute of Medical Sciences-Bhubaneswar, Bhubaneswar, Orissa, India; 4 St George's University of London, London, UK

**Keywords:** Congenital Abnormalities, Data Collection, Child Health, Epidemiology, Ethology

## Abstract

**Background:**

Polyglactin (PG) and polydioxanone (PDS) sutures are extensively used based on the surgeon’s preference. The development of post-reconstruction urethrocutaneous fistula (UCF) is variably attributed to the choice of suture material for urethroplasty. This meta-analysis compares complications of hypospadias repair using PG and PDS sutures.

**Methods:**

The systematic review and meta-analysis were performed as per the Preferred Reporting Items for Systematic Reviews and Meta-Analyses guidelines. The authors conducted thorough searches in databases including MEDLINE, EMBASE, CENTRAL, Scopus, Google Scholar, and clinical trial registries. Outcome measures included UCF, meatal stenosis, wound infection, urethral stricture, glans dehiscence, and overall complications. Quantitative analysis was used with fixed or random-effect models to find the pooled risk ratio and I^2^ heterogeneity.

**Results:**

The criteria for inclusion were met by five comparative studies with the inclusion of 1244 children altogether. Pooled analysis failed to show a statistically significant difference in the incidence of meatal stenosis, urethral stricture, wound infection, and total complications using PG and PDS sutures. However, it showed a reduction in the incidence of UCF with PDS suture hypospadias repairs (risk ratio=0.66, 95% CI 0.48 to 0.92).

**Conclusions:**

PDS sutures are associated with decreased incidence of UCF than PG after hypospadias repair. The incidence of meatal stenosis, urethral stricture, wound infection, and total complications was not affected by the type of suture material used for repair.

**Clinical implications:**

This meta-analysis suggests decreased incidence of UCF when PDS sutures are used for hypospadias repair which may impact the choice of suture material for repair.

**PROSPERO registration number:**

CRD42023409710.

**WHAT IS ALREADY KNOWN ON THIS TOPIC**

- The development of post-reconstruction urethrocutaneous fistula (UCF) is a matter of concern even with many technical advances over the years.
- The use of absorbable sutures for hypospadias surgery is widely agreed upon.
- Among these, the polyglactin and polydioxanone (PDS) are commonly used sutures based on the individual surgeon’s preference.

**WHAT THIS STUDY ADDS**

- This quantitative analysis shows decreased incidence of UCF when PDS sutures are used for hypospadias repair. The choice of suturing material may affect the incidence of UCF during hypospadias repair.
- The incidence of meatal stenosis, urethral stricture, wound infection, and total complications, however, was not affected by the type of suture material used in hypospadias repair.

**HOW THIS STUDY MIGHT AFFECT RESEARCH, PRACTICE OR POLICY**

- The results of this study could impact the decision-making process in hypospadias repair when selecting suture materials for clinical practice.
- The clinical heterogeneity among the included studies created further domains of standardized and controlled research in this area.

## Introduction

Hypospadias is one of the most frequent birth defects affecting the male urethra. The prevalence of hypospadias varies across countries ranging from 2.1 to 39.1 per 10 000 live births.^1^ Aberrant ventral placement of the urethral meatus is characteristic of hypospadias. The anomalous urethral meatus can be located anywhere between the perineum and the tip of the glans penis. Depending on the anatomical location of the meatus, hypospadias can be categorized as glanular, penile, proximal, and perineal.^2^ The Hypospadias International Society recommends the meatus–chordee–glans width and shape-urethral plate quality score to describe the severity of hypospadias. The score ranges from 0 (normal) to 10 (perineal hypospadias with severe chordee, small glans, and narrow urethral plate).^2^ The English literature describes more than 300 methods of hypospadias repair.^3^ Satisfactory reconstruction should provide a cosmetically pleasant straight phallus with a vertically slit meatus at the glans tip. Despite technical developments such as the use of magnification and improvisation of the surgical equipment, urethrocutaneous fistula (UCF) remains the most frequent complication following hypospadias correction. The occurrence of UCF ranges from 7.5% to 50%, depending on the surgical repair technique and the severity of hypospadias.^4^ The UCF can develop anywhere across the length of the repair. Creating an intermediate protective layer to cover the neourethra has reduced the incidence of UCF in hypospadias repair. This protective intermediate layer can be fashioned using the de-epithelialized local skin, tunica vaginalis flap, local dartos, and free tunica vaginalis graft.^5–8^ Delicate tissue handling, use of bipolar diathermy, and microsurgical techniques also reduced the incidence of UCF in these patients.

One of the key factors affecting the complications following hypospadias repair can be the choice of the suture material. The use of absorbable sutures for hypospadias surgery is widely agreed upon among surgeons. However, choosing any particular suture material has always been a matter of individual preference. The ideal suture material used for neourethra construction should provide sufficient mechanical support and resist the chemical effect of urine until satisfactory wound healing. Polyglactin (PG) and polydioxanone (PDS) are the two widely used absorbable sutures for hypospadias repair. PG is a synthetic, braided, absorbable suture made of a copolymer of glycolide and lactide. It has a predictable and reliable absorption rate, typically lasting 4–6 weeks. PG is widely available, easy to handle, has good knot security, and is cost-effective. The drawback is intense tissue inflammation, higher capillarity, and tissue drag and trauma by its rough surface.^9^ PDS is another synthetic, absorbable suture made of a monofilament polymer of p-dioxanone. It has a slower absorption rate, lasting up to 6 months, which may provide more long-term wound support. PDS sutures are preferable for repairing delicate and high-tension wounds due to minimum tissue reaction, lower tissue drag, and minimal capillarity leading to fewer suture-related complications.^9^ On the other hand, they are more expensive than PG and may be less widely available.

Few studies indicated that PDS might not be suitable for urethral and penile reconstruction but other studies found that it provides better tissue support for wound healing. Guarino *et al.*, in a comparative study of suture materials in hypospadias repair, observed a higher rate of pinpoint fistulae in the PDS group on long-term observation.^10^ The development of UCF was attributed to the prolonged absorption time of PDS. On the contrary, Ulman *et al.* reported a lower complication rate with subcuticular PDS compared with interrupted suturing with PG in hypospadias repair.^11^ Other studies that compared PDS and PG in hypospadias repair also failed to reach a precise conclusion. Studies by Khalid *et al.* 9 and Cimador *et al.* 12 reported no discernible variation in the frequency of UCF while using PG and PDS. Mohamed Ali Alaraby *et al.*,^13^ Fakhr,^14^ and Shizari *et al.* 15 reported reduced complications with PDS. The inconsistent outcomes of the comparative studies on the choice of suture materials in hypospadias repair led to the planning of this systemic review and meta-analysis. This meta-analysis intends to evaluate postoperative complications following a urethroplasty using PG or PDS during hypospadias repair.

## Methods

### Search strategy

This systematic review and meta-analysis was registered in the PROSPERO database (CRD42023409710). The systematic review and meta-analysis were performed as per the PRISMA (Preferred Reporting Items for Systematic Reviews and Meta-Analyses) guidelines.^16^ Databases including MEDLINE, EMBASE, Scopus, and the Cochrane Central Register of Controlled Trials were searched for relevant studies. The WHO International Clinical Trials Registry Platform and US National Institutes of Health Ongoing Trials Register (ClinicalTrials.gov) were additionally screened for unpublished but completed research.

Randomized and comparative studies evaluating the outcome of hypospadias repair with PG and PDS for the meta-analysis with full-text articles published in the English language were included. Unpublished data from finished studies available from gray literature were also included. (Gray literature is the information produced outside of traditional publishing and distribution channels. In can include reports, policy literature, working papers, newsletters, government documents, speeches, etc).

The search terms used were (polyglactin OR vicryl OR polyglactin 910 OR PDS OR polydioxanone) AND (hypospadias repair OR hypospadias surgery OR urethroplasty). A citation search was performed by forwarding citations of studies on Google Scholar on included studies to identify new studies. Searches were conducted up to April 10, 2023, and rerun before the final analysis. Two authors (CKS and NB) completed the study searching and review. Any discrepancies between the authors were settled by mutual discussion, and a third author (CKS) was consulted in case of lack of consensus.

### Data extraction and management

The study characteristics that were documented included author details, year of publication, year and place of conduct, study period, number of patients, mean age of participants, study design (eligibility criteria, randomization, and follow‐up period), suture material, and suturing technique. The primary outcomes were UCF, glans dehiscence, meatal stenosis, urethral stricture, wound infection, and total complications. The Downs and Black Scale was used for evaluating the methodological quality of included studies.^17^ The quality ratings assigned to the ranges of Downs and Black Scale scores were excellent (26–28), good (20–25), fair (15–19), and poor (<14).

### Statistical analysis

Data were analyzed using Review Manager (RevMan V.5.4; The Cochrane Collaboration, 2020). Continuous variables were analyzed as mean differences with 95% CIs using an inverse variance approach. Dichotomous variables were analyzed as risk ratios (RRs) with 95% CIs using the Mantel-Haenszel technique. The results were compared with fixed and random-effect models. When p>0.01 and I^2^<50%, a fixed-effect model was used, and when p<0.01 and I^2^>50%, a random-effect model was used.^18^

## Results

### Study characteristics

**Figure 1:**
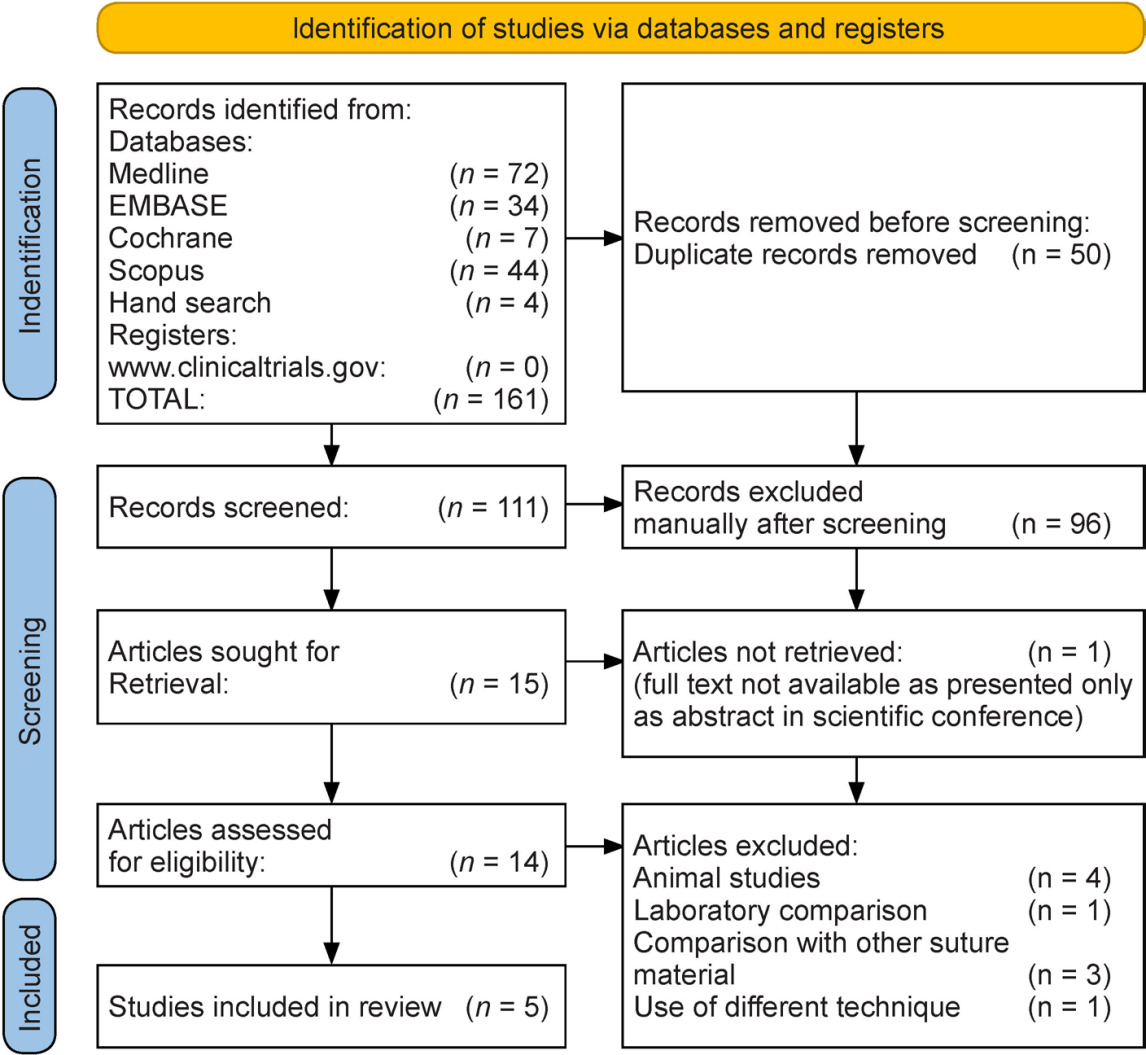
Preferred Reporting Items for Systematic Reviews and Meta-Analyses flow diagram.

Following the literature search, a total of 161 studies were identified. The search and selection procedure is depicted in the PRISMA flow diagram (figure 1). A total of 111 records were examined by title and abstract after the elimination of duplicate studies. Fifteen articles were shortlisted for full-text screening, of which only five research articles^9 12–15^ were considered for the final quantitative analysis. The study characteristics of the included studies are summarized in table 1. The five studies included 1244 children, with 532 in the PDS group and 712 in the PG group. The study outcomes are summarized in table 2.

**Table 1:** Characteristics of included studies

S/N	Studies	Setting	Study period	Design	Patients (n)	Mean age (months)	Type of hypospadias	Suturing technique and materials	F/U (months)	Reported outcomes
1	Cimador *et al.* 12	Italy	1991–2001	Bidirectional	336 (254 in group A and 82 in group B)	Not mentioned	Subcoronal Distal Mid-shaft Proximal	Group A: PG 6/0 (254 patients) Group B: PDS 7/0 (82 patients) Continuous subcuticular Second layer: dartos flap	Not mentioned	UCF US
2	Khalid *et al.* 9	Pakistan	2009–2016	Multicentric RCT	200 (100 in each group)	Group A: 3.7±1.8 years (1.6–9.6 years) Group B: 3.6±1.6 years (1.8–8.9 years)	Anterior hypospadias	Group A: PG 6/0 (100 patients) Group B: PDS 6/0 (100 patients) Interrupted subcuticular Second layer: dartos flap	6 months	UCF WI MS UCF+MS
3	Shirazi *et al.* 15	Iran	January 2012–December 2018	Cross-sectional study (bidirectional)	583 (298 in group A and 285 in group B)	Complicated group: 3±1.32 years Uncomplicated group: 3.1±1.15 years	Distal Mid-shaft Proximal	Group A: PG 6/0 (298 patients) Group B: PDS 6/0 (285 patients) TIP Second layer: dartos flap TVS	2 years	UCF MS US GD
4	Mohamed Ali Alaraby *et al.* 13	Sudan	June 2015–November 2016	Prospective cross-sectional comparative study	105 (50 in group A and 55 in group B)	Group A: 5.7±4.3 years Group B: 5.1±3.9 years	Distal Mid-shaft Proximal Perineal	Group A: PG 6/0 (50 patients) Group B: PDS 6/0 (55 patients) Second layer: dartos flap	6 months	Overall complications UCF MS
5	Fakhr^14^	Lahore	2018 (6 months)	Comparative prospective study	20 (10 in each group)	5.4 years (2–11 years)	Anterior hypospadias	Group A: PG 6/0 (10 patients) Group B: PDS 6/0 (10 patients)	6–9 months	Total complications Erythema Edema Bleeding UCF WI

F/U, follow-up; GD, glans dehiscence; MS, meatal stenosis; PDS, polydioxanone; PG, polyglactin; RCT, randomized controlled trial; TIP, tubularized incised plate; UCF, urethrocutaneous fistula; US, urethral stricture; WI, wound infection.

**Table 2:** Summary outcomes

	Study	Technique	Type of repair	Total (n)	UCF	MS	Stricture	GD	WI	Redness/ erythema	Bleeding	Swelling/edema	Flap necrosis	Total complications
1	Cimador *et al.* 12	PG	Mathieu technique: 277 patients (subcoronal hypospadias) Transverse preputial flap (tubularized or onlay): 59 patients (midshaft or proximal hypospadias)	254	17	–	12	–	–	–	–	–	–	29
		PDS		82	6		2				–	–	–	8
2	Khalid *et al.* 9	PG	Snodgrass repair of subcoronal hypospadias	100	29	6	–	–	7	–	–	–	–	42
		PDS		100	26	9	–	–	4	–	–	–	–	39
3	Fakhr^14^	PG	Only anterior hypospadias (surgery not mentioned)	10	0	–	–	–	5	4	9	5	3	26
		PDS		10	0	–	–	–	2	3	5	0	0	10
4	Shirazi *et al.* 15	PG	All underwent TIP with dartos flap for distal, mid-penile, and TVS flap for proximal hypospadias	298	27	8	6	4	–	–	–	–	–	45
		PDS		285	12	2	1	0	–	–	–	–	–	15
5	Mohamed Ali Alaraby *et al.* 13	PG	Single-stage repair: 79.3% Multistage repair: 20.7%. MAGPI: 47.6% TIP: 31.4% Thiersch-Duplay: 20% Mathieu repair: 1%	50	14	9	–	–	2	–	0	0	–	17
		PDS		55	5	2	–	–	2	–	4	11	–	6

GD, glans dehiscence; MAGPI, meatal advancement and glanduloplasty; MS, meatal stenosis; PDS, polydioxanone; PG, polyglactin; TIP, tubularized incised plate; UCF, urethrocutaneous fistula; WI, wound infection.

### Methodological quality assessment of included studies

The modified Down and Black Scale scores of the included studies ranged from 16 to 23. The study by Khalid *et al.* 9 received the best score, whereas the study by Cimador *et al.* 12 received the lowest score. Raters 1 and 2 had a strong, positive correlation (κ=0.99), indicating almost perfect agreement between both raters.

### Meta-analyses of the complications

#### Urethrocutaneous fistula

**Figure 2:**
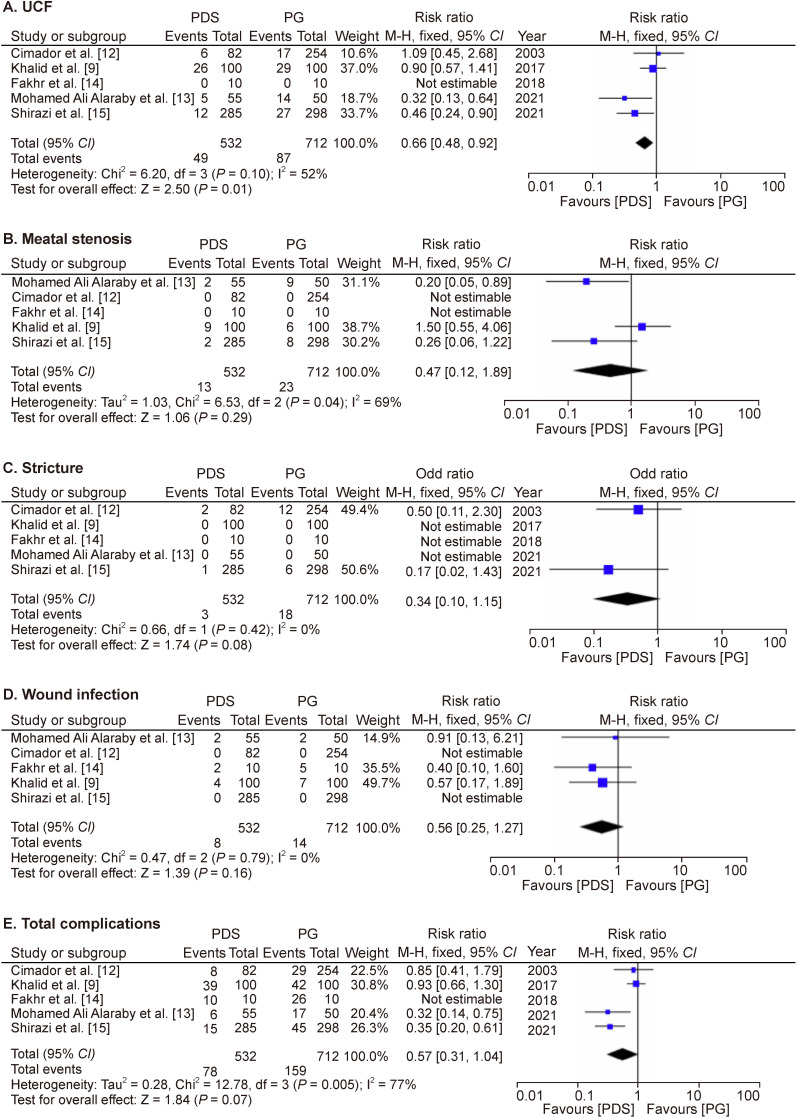
Forest plots depicting: (A) Urethrocutaneous fistula; (B) Meatal stenosis: (C) Stricture; (D) Wound infection; (E) Total complications. PDS, polydioxanone; PG, polyglactin; UCF, urethrocutaneous fistula.

Four studies have described this complication. There were 49 (9%) UCFs among 532 patients with PDS, while 87 (12%) UCFs were identified among 712 patients in the PG group. Pooled analysis (figure 2A) of this complication shows a statistically significant reduction in the incidence of UCF with PDS compared with PG, though with moderate heterogeneity (p=0.01, RR=0.66, 95% CI=0.48 to 0.92, I^2^=52%).

There is heterogeneity in the technique of hypospadias repair among the included studies. Heterogeneity also exists in the type of hypospadias. Studies by Khalid *et al.* 9 and Fakhr^14^ included only anterior hypospadias. In contrast, Cimador *et al.*,^12^ Mohamed Ali Alaraby *et al.*,^13^ and Shirazi *et al.* 15 included all types of hypospadias for the comparative analysis. Cimador *et al* 12 used Mathieu transverse preputial tubularized flap and onlay technique, while Khalid *et al.* 9 and Shirazi *et al.* 15 used the Snodgrass technique to repair hypospadias. Mohamed Ali Alaraby *et al.* 13 used meatal advancement and glanduloplasty, Mathieu, tubularized incised plate (TIP), and Thiersch-Duplay techniques for hypospadias repair. Fakhr^14^ did not mention the repair technique in their patients. This heterogeneity among the different studies may be insignificant as there is no heterogeneity among the PDS and PG groups in the included studies.

We conducted a subgroup analysis of the studies that exclusively used the TIP technique for the outcome analysis of UCF. Khalid *et al.* 9 and Shirazi *et al.* 15 used the TIP technique to repair all their included patients. Subgroup analysis in these studies shows no statistical difference among the studied variables (RR=0.69, 95% CI=0.48 to 1.00).

#### Meatal stenosis

Only three studies included in the meta-analysis reported this outcome. The pooled analysis (figure 2B) of these three studies revealed no discernible difference in the rate of meatal stenosis between the PDS and PG groups, with substantial heterogeneity (p=0.29, RR=0.56, 95% CI=0.29 to 1.08, I^2^=69%).

#### Stricture

Only two studies included in the meta-analysis reported this complication. A pooled analysis (figure 2C) of the two studies failed to show a statistical difference in the stricture rate between the PDS and PG groups without heterogeneity (p=0.08, RR=0.34, 95% CI=0.10 to 1.15, I^2^=0%).

#### Wound infection

Only three included studies in the meta-analysis reported this complication. Pooled analysis (figure 2D) of the three studies showed no significant difference in the wound infection rate between the PDS and PG groups (p=0.16, RR=0.56, 95% CI=0.25 to 1.27, I^2^=0%) without heterogeneity.

#### Total complications

All five studies included in this meta-analysis reported this outcome. The reported incidence of total complications outnumbered the included patients in the study by Fakhr.^14^ The development of multiple complications in a single patient in this study is a plausible explanation. We analyzed it as per the number of patients who developed the complications; however, we excluded the study from the analysis of this outcome. Khalid *et al.* 9 reported that all patients with wound infection also had UCF, so we calculated the total number of patients with complications excluding this number. Pooled analysis (figure 2E) of the remaining four studies showed no statistically significant difference in the incidence of total complications among the PDS and PG groups (p=0.07, RR=0.57, 95% CI=0.31 to 1.04, I^2^=77%) with substantial heterogeneity.

#### Glans dehiscence

This outcome was reported by only one study, so pooled analysis was not done.

## Discussion

The present systematic review revealed a decreased incidence of UCF among the pediatric patients in whom PDS was used for the hypospadias repair. The other outcome variables such as meatal stenosis, wound infection, stricture rate, and total complications were comparable in both PDS and PG groups.

Suture choice for hypospadias repair depends on factors like absorption time, tensile strength, and surgeons’ preference based on their experience. Various researchers have debated the ideal absorbable suture material for hypospadias repair. Different suture materials have been studied in vivo and in vitro for their suitability for hypospadias repair. Shirazi *et al.* 19 studied the effect of different types of absorbable suture materials for hypospadias correction in a rat model. They found chromic catgut, poliglecaprone 25, PG 910, and polylactic acid suitable for urethroplasty. In the PDS group, the volume of the urethral epithelium was found to be higher compared with other suture materials. Bartone *et al.* 20 studied chromic catgut, PDS, and polyglycolic acid (Dexon) under similar conditions in the penile foreskin of baboons. They concluded that chromic catgut is the best suture for use in the penile foreskin. Polyglycolic acid and PDS should be avoided in hypospadias repair because of their prolonged resorption and excess tissue reaction.^20^ A porcine model study on UCF by Edney *et al.* 21 and Lopes *et al.* 22 found that the suture tracks begin to develop from day 5, and a mature fistula is found around day 12. Therefore, the ideal suture material should have a minimum tensile strength of 5 days and degrade by 12 days before the suture tracks are established, but this quality is lacking in PDS. The suture material is also required to hold the wound together until the healing tissue is strong enough to prevent breakdown. The major drawback of all the above studies was that they were conducted in animal models with no histological resemblance to the human urethra.

The effects of human urine on the tensile strengths of 6/0 Vicryl, Vicryl Rapide, PDS, and Monocryl sutures commonly used in hypospadias surgery were investigated by Kerstein *et al.* 23 Vicryl was the best suture for hypospadias repair which balanced the dangers of foreign body reaction with sufficient duration of support for the healing surgical incision. DiSandro and Palmer^24^ evaluated the stricture incidence in hypospadias surgery using sutures like PDS, chromic catgut, and polyglycolic acid. They found that PDS had a three to four times higher incidence of stricture formation than chromic catgut and polyglycolic acid. They proposed that the best results in hypospadias correction come from sutures with mid-range absorption rates.^24^ The delayed in vivo absorption rate of PDS should preclude its use in urethral anastomosis.^24^ Guarino *et al.* 10 compared the results of hypospadias using PG and PDS. They found no statistically significant differences among both sutures regarding the development of UCF. Their long-duration follow-up study detected a greater incidence of pinpoint fistulae in the PDS group. They attributed the prolonged persistence of PDS and subsequent epithelialization of the suture track to fistula formation. However, they concluded that both suture types are suitable for hypospadias surgery.

Though the use of Vicryl seems ideal based on the studies mentioned above, it is a polyfilament suture at risk of inducing severe local inflammation. It also has the highest bacterial adherence compared with other monofilament sutures like Monocryl.^25^ Wound infection may act as a nidus leading to the development of UCF. Delicate tissue handling, magnification, and tension-free repair are the strategies against fistula prevention. PDS has been used worldwide by many hypospadias surgeons over the years with comparable rates of complications. Ulman *et al.* 11 were the first to compare PDS and PG; they observed that subcuticular PDS sutures were associated with a lower complication rate. This study was excluded from our meta-analysis due to a difference in the suturing technique, which can act as a confounding factor for UCF development as per a recent meta-analysis.^26^ In our quantitative analysis, there is heterogeneity in suturing technique among the different studies but not among the PDS and PG groups. Almost all studies using animal models had discouraged PDS for hypospadias repair to minimize the incidence of UCF; however, our meta-analysis shows a decreased incidence of UCF with PDS. Our meta-analysis also raises a question about the validity of animal studies for hypospadias repair. A recent publication by Yamashiro *et al.* 27 has discussed the various suture materials in the variability of surgical practice and instrumentation used for hypospadias repair. In their analysis, PG stands as the first choice as suture material among surgeons, followed by PDS, which also substantiates the need for our quantitative analysis on choice of suture material.

### Limitations

This meta-analysis has a high clinical heterogeneity among the included studies concerning the severity of hypospadias and surgical techniques. In these studies, all types of hypospadias were repaired using PDS and PG. However, we could not ascertain whether the difference in the number of types of hypospadias among the PDS and PG groups was statistically significant or not. Therefore, it is necessary to conduct randomized controlled studies using PDS and PG for hypospadias repair with larger sample sizes, comparable patient features, and surgical techniques.

### Conclusion

The choice of suturing material may significantly affect the incidence of UCF during hypospadias repair. Our meta-analysis shows a decreased incidence of UCF with PDS in hypospadias repair; however, the severity of the hypospadias and the type of surgical repair can be the confounding factors influencing this outcome. No differences in other complications like meatal stenosis, stricture, wound infection, and total complications were found.

## Data Availability

All data relevant to the study are included in the article or uploaded as supplemental information.
